# Oral Toxicity Profile of a Novel Silk Lutein Extract and Assessment of Its Cardiovascular Benefits in Rodents

**DOI:** 10.3390/ijms27020577

**Published:** 2026-01-06

**Authors:** Chainarong Tocharus, Manote Sutheerawattananonda

**Affiliations:** 1 Department of Anatomy, Faculty of Medicine, Chiang Mai University, Chiang Mai 50200, Thailand; chainarongt@hotmail.com; 2 School of Food Technology, Institute of Agricultural Technology, Suranaree University of Technology, Nakhon Ratchasima 30000, Thailand

**Keywords:** yellow silk lutein, *Bombyx mori*, oral toxicity, safety evaluation, hypotensive effect, nutraceutical

## Abstract

Silk Lutein (SL), a novel protein-bound lutein complex derived from *Bombyx mori* cocoons, offers an intriguing alternative to traditional sources. This study aimed to establish the complete toxicological profile of SL. Evaluations of the acute oral toxicity of SL (LD_50_ > 2000 mg/kg body weight (BW)) were conducted in female Wistar rats and ICR mice. In the chronic toxicity trial, male and female Wistar rats were administered daily oral dosages of SL (5, 25, 50 mg/kg BW) for a duration of six months. The results indicated a robust safety profile for SL, with no treatment-related adverse effects detected. Apart from demonstrating its foundational safety, this study found that prolonged SL administration possessed significant, beneficial bioactive properties. Following four months of treatment, both male and female Wistar rats administered SL exhibited a significant hypotensive effect, maintaining their systolic blood pressure at approximately 120 mmHg and thereby averting the age-related hypertension observed in control subjects. Additionally, SL significantly reduced serum triglyceride levels in both sexes. The findings of this study confirm SL’s potential as a multipurpose nutraceutical by demonstrating that it is a safe constituent with a favorable toxicological profile and notable cardiovascular effects.

## 1. Introduction

As the world’s population continues to age, the prevalence of age-related chronic diseases has also increased. Age-related macular degeneration (AMD) is one of the main causes of permanent blindness [1]. This has spurred an urgent search for effective preventive strategies, positioning nutritional science at the forefront of public health interventions. Within this paradigm, the xanthophyll carotenoid lutein has been identified as a critical protective micronutrient. Since lutein cannot be produced by the body, it is a necessary nutrient that must be consumed through food, such as egg yolks, corn, and leafy green vegetables [2]. Its selective accumulation in the macula, where it forms the macular pigment, is its primary physiological significance. This provides a dual defense mechanism by serving as a precise filter for phototoxic high-energy blue light and as a strong localized antioxidant [3,4]. The Age-Related Eye Disease Study 2 (AREDS2) provided definitive evidence that daily supplementation markedly decreases the risk of progression to advanced age-related macular degeneration (AMD), thereby establishing lutein supplementation as a clinical standard and contributing to a growing global market [5].

Marigold flower (*Tagetes erecta*) extracts have been the primary source of this industrial requirement for decades [6]. Nonetheless, the marigold paradigm is limited by the intrinsic chemical instability of lutein, which is especially vulnerable to degradation due to factors including heat, light, and oxygen, posing considerable challenges in preserving potency across the supply chain [6,7]. Previous research has focused on increasing lutein stability via encapsulation, chemical bonding with protein and fatty acids, with some successes [8,9,10]. However, refrigeration and deep freezing are needed during logistics and long-term storage, which is not practical in the hot and humid regions like Southeast Asia, Africa, and Latin America. This has spurred a scientific quest for a next-generation lutein source that offers superior stability. An exceptionally promising candidate has emerged from the golden-yellow silk cocoons of the *Bombyx mori* silkworm that has been reared for local silk production in Thailand for a century. The lutein within these cocoons, SL, is not a free molecule but is naturally and intricately bound within a sericin protein matrix [11,12]. This protein-bound configuration structurally mimics the form of lutein in the human macula, where it is also bound to specific proteins to ensure stability and function [13,14]. According to recent data, the only known structure found naturally in yellow silk cocoons is a special, naturally sericin-bound form that offers significantly better thermal and oxidative stability than commercial lutein [15].

Apart from the possibility of improved stability, lutein has been linked to more extensive systemic health advantages. Due mainly to its antioxidant qualities, which en-67 hance nitric oxide (NO) bioavailability and preserve endothelial function, lutein has been 68 shown in animal models to have a hypotensive impact [16]. This is particularly relevant as vascular dysfunction is a shared pathophysiological feature in both AMD and hypertension. Previous studies have shown that SL exhibit the ability to protect PC12 cells against β-amyloid peptide-induced mortality [17], human keratinocytes from UV-B damage [18], and retinal pigment epithelial cells from the detrimental effects of H_2_O_2_ [19]. It has been previously documented that SL has ocular and immunomodulatory benefits [20]; however, the effects on the cardiovascular system in vivo and, most importantly, the whole toxicological profile of this new protein-bound lutein extract have never been demonstrated.

Given the unique protein-bound nature of SL, which may confer enhanced stability and bioactivity, this study was designed to address two critical knowledge gaps. The primary objective was to conduct the first comprehensive toxicological evaluation of the SL complex of its acute and chronic safety profile in a single dose of 2000 mg/kg BW and a six-month rodent study, respectively. Investigating whether long-term administration of this novel, protein-bound lutein form would result in any significant, value-added physiological benefits was the secondary, exploratory goal. Key cardiovascular health indicators like blood pressure and serum lipids were specifically addressed.

## 2. Results and Discussion

### 2.1. Characterization of the SL Extract

The SL extract utilized in this study was a dark yellow, lipid-soluble dried extract. The complete specifications of the SL extract are presented in Table 1. As analyzed by gas chromatography, the SL extract contained 94.84% *w*/*w* fat, while the remainder was insoluble unidentified wax materials. Chemical analysis confirmed that the SL extract consisted of a complex lipid matrix with 4.12–6.35% lutein. The remaining portion of the extract was primarily crude fat, consistent with findings previously reported by Promphet et al. (2014) [20].

Crucially, the safety profile of the SL extract was rigorously established. The levels of trace metals, including lead (Pb), cadmium (Cd), and arsenic (As), were all found to be well below the maximum permissible limits for food-grade ingredients set by the Thai Ministry of Public Health’s Announcement No. 414 (2020) [21], except mercury (Hg). Lutein is recommended to be taken daily at 6 to 14 mg to reduce more than 50% risk of AMD [22]. Lutein dietary intake would provide 0.088–0.137 μg of Hg daily, in case of SL. This level is roughly 15 times lower than the reference dose of 2 μg per day recommended by the Environmental Protection Agency (EPA) to protect human health [23]. Table 2 summarizes this data. Microbiological analysis confirmed the extract’s safety, with total plate counts, yeast and mold, *E. coli*, and *Salmonella* spp. all meeting the standards for materials intended for oral consumption. Furthermore, no residues of key pesticide classes, including organochlorines and organophosphates, were detected. These characterization results confirm the SL batch’s safety for the ensuing toxicological analyses.

**Figure 1:**
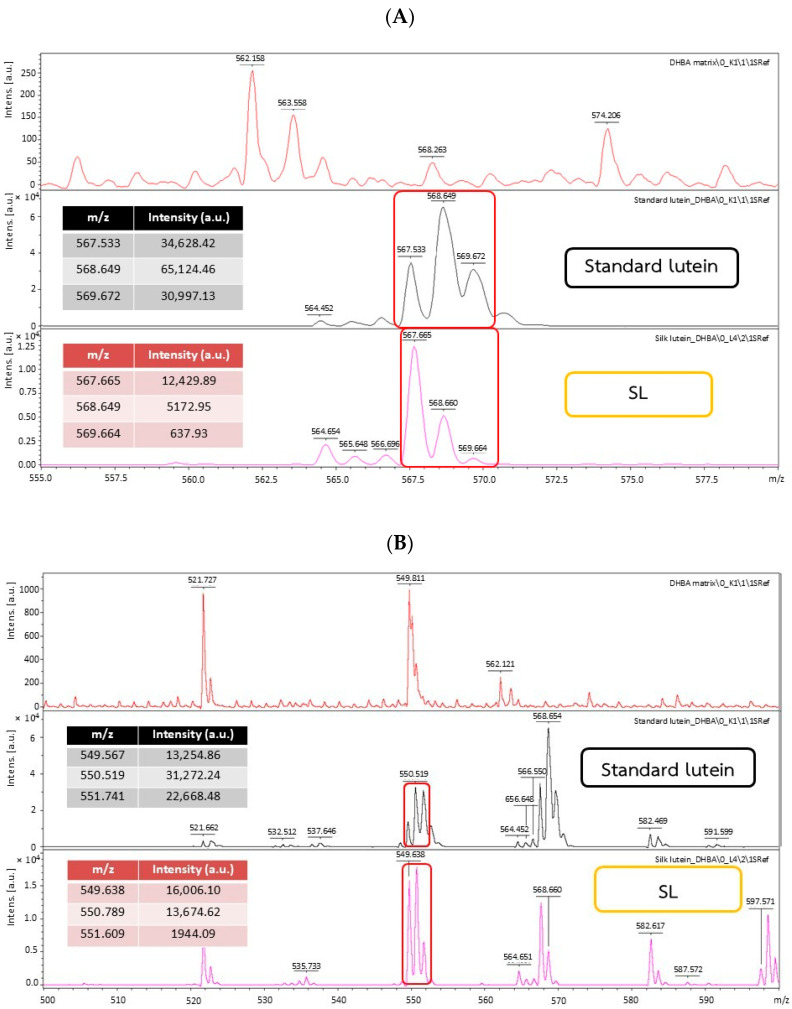
Mass spectra of standard lutein and SL extracted from yellow silk cocoons in the *m*/*z* range of 555–580 (**A**) and 550–600 (**B**).

Figure 1 presents the comparative absorption spectra of the SL extract and an authentic lutein standard. The structural identity of the compound isolated from yellow silk cocoons was confirmed via mass spectrometry by comparing its spectral signature with that of an authentic lutein standard. The mass spectra of the SL extract exhibited characteristic ion peaks that aligned closely with those of the standard. Figure 1A highlights the molecular ion region, where the standard displayed a dominant peak at *m*/*z* 568.649, corresponding to the molecular mass of lutein. The SL extract showed a matching signal cluster with high intensity around *m*/*z* 567–568. Furthermore, Figure 1B reveals a consistent fragmentation pattern in the *m*/*z* 550 range for both samples (Standard lutein: *m*/*z* 550.519; SL: *m*/*z* 549.638). The identity of these spectral fingerprints, specifically the matching peak distributions highlighted in the red boxes, provides strong evidence that the pigment extracted from the silk cocoons is structurally identical to lutein.

Table 3 details the specific mass-to-charge ratios (*m*/*z*) and intensities derived from the LC-MS analysis, providing quantitative support for the spectral alignment shown in Figure 1. The data reveal a precise correspondence between the standard and SL extract, particularly regarding the molecular ion, which appears at *m*/*z* 568.6 for the standard and *m*/*z* 568.7 for SL. This numerical agreement corroborates the visual evidence in the mass spectra, confirming that the primary compound in the silk extract is structurally identical to authentic lutein.

**Figure 2:**
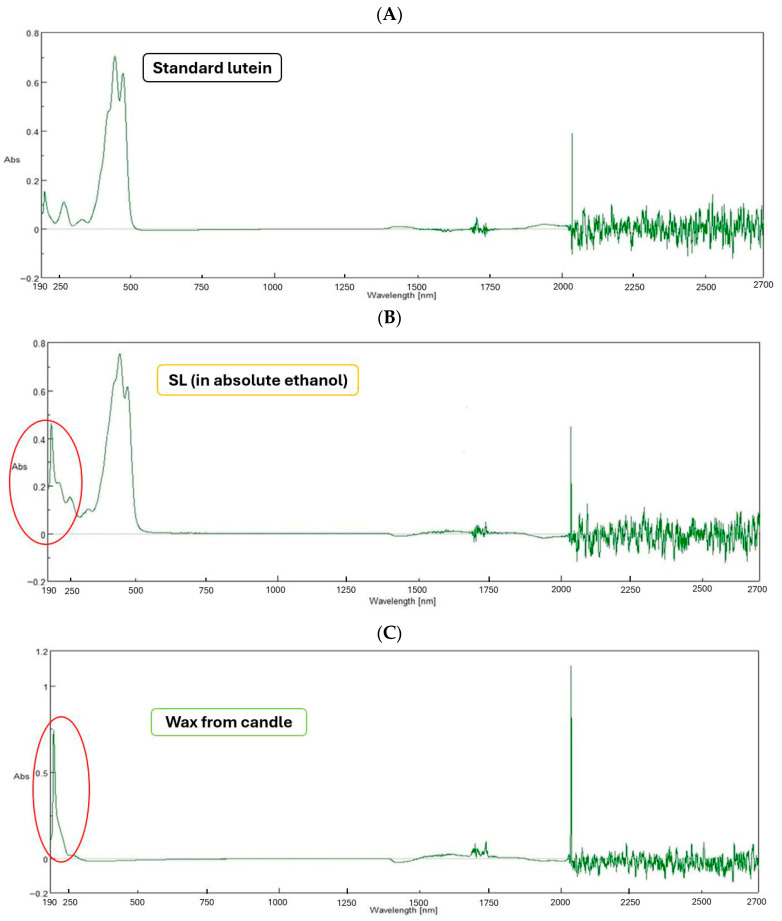
UV-Vis absorption spectra of standard lutein (**A**), SL from yellow silk cocoons (**B**), and wax from a candle (**C**), recorded across the wavelength range of 190–2700 nm. The red circles highlight the spectral similarity in the UV region (190–250 nm) between the SL extract and the wax standard, indicating the presence of waxy impurities in the extract.

The UV-Vis absorption spectra confirm the identity of the compound extracted from yellow silk cocoons while simultaneously characterizing its purity profile. As shown in Figure 2, the SL extract exhibited a characteristic absorption profile in the visible region (400–500 nm) that closely mirrored that of the standard, displaying the distinct three-peak vibronic structure typical of lutein (with maxima at approximately 425, 446, and 474 nm). This spectral fingerprint confirms the presence of lutein in the extract. However, a notable difference was observed in the ultraviolet region (190–250 nm), where the silk extract displayed significant absorbance (highlighted in the figure) that was absent in the pure standard. Crucially, this distinct UV absorption pattern closely resembles the spectrum obtained from candle wax. This spectral similarity strongly suggests that the co-extracted impurities are likely waxy substances inherent to the silk cocoon matrix, such as sericin-associated waxes or lipids, rather than generic proteins. These waxy components absorb strongly at lower wavelengths but do not interfere with the characteristic lutein signature in the visible range.

**Figure 3:**
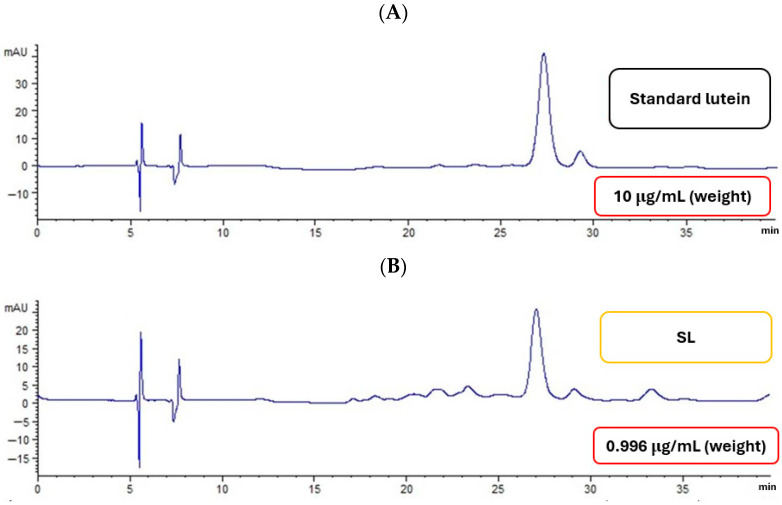
Chromatographic profiles of standard lutein (**A**) and SL from yellow silk cocoons (**B**) at 450 nm.

HPLC analysis confirmed the identity and content of the lutein extracted from yellow silk cocoons by comparing its retention time and quantification from the lutein standard curve with that of a standard lutein (Figure 3). The results indicated that the extract contained 3.50–5.40% *w*/*w* lutein. The resulting chromatograms for both the silk extract and the standard lutein are shown in Figure 3. As illustrated in the chromatograms, the major peak in the silk extract eluted at approximately 27 min, aligning perfectly with the retention time of the standard lutein; this coincidence definitively identifies the primary compound in the extract as lutein. However, the chromatographic profiles also reveal differences in purity between the two samples. While the standard displayed a clean profile dominated by a single major peak, the SL chromatogram exhibited several minor peaks eluting between 15 and 25 min. These additional signals likely represent structural isomers, other minor carotenoids, or matrix components co-extracted from the cocoon, indicating that the silk extract is a complex mixture compared to the highly refined standard.

### 2.2. Acute Oral Toxicity Study

The acute oral toxicity of the novel SL extract was evaluated in a limit test design. A single administration of SL at a dose of 2000 mg/kg BW did not produce any mortality or treatment-related clinical signs of toxicity in either female Wistar rats or female ICR mice over the 14-day observation period. This finding establishes the Median Lethal Dose (LD_50_) of SL to be greater than 2000 mg/kg BW in both rodent species, classifying it as a substance with low acute toxicity potential [29]. Further supporting this conclusion, there were no statistically significant differences in body weight gain or the absolute weights of internal organs between the SL-treated and control groups (Table 4). This lack of effect on key physical parameters underscores the non-toxic nature of SL at high-dose administration.

#### 2.2.1. Clinical Chemistry

Analysis of clinical chemistry parameters revealed several statistically significant, species- and sex-dependent variations, though none were considered toxicologically adverse (Table 5). When SL was administered to female Wistar rats, ALP significantly increased, whereas creatinine and ALT were decreased. In female ICR mice, SL treatment led to a significant increase in BUN and cholesterol, and a significant decrease in ALT. While these variations reached statistical significance, their toxicological relevance must be interpreted within the context of normal biological variability. Due to the restricted total blood volume available from individual mice (typically <1 mL), which limits the breadth of an exhaustive biochemical panel, these parameters were cross-referenced against established historical control data and published normal physiological ranges for the ICR strain [30]. Since ALT leaks from the cytoplasm of damaged hepatocytes into the bloodstream, its rise in serum is a typical sign of liver cell destruction. ALT is a highly specific biomarker for hepatocellular integrity [31]. The observed reduction in ALT contradicts a toxicological signal. A decrease in this enzyme is usually considered a benign or potentially advantageous physiological phenomenon rather than an indication of liver damage [32]. This finding strongly suggests that SL, even at a high dose, does not induce hepatocellular injury.

This conclusion is further supported by the fact that this impact is consistent across two distinct rodent species. There are a few possible interpretations for the observed decrease. It could indicate a change in amino acid metabolism that lowers baseline enzyme levels, a possible hepatoprotective effect by stabilizing hepatocyte membranes, or an improvement in basal liver health [33].

Serum ALP levels were significantly elevated in female Wistar rats treated with SL, increasing from 85.50 to 114.67 U/L, in contrast to ALT levels. In toxicology, a substantial increase in ALP could be a symptom of biliary epithelial damage or cholestasis (disrupted bile flow) [34]. This diagnosis is typically only made, though, when the increase in ALP is accompanied by increases in ALT and AST, as well as other markers of cholestatic damage like bilirubin [35]. In our study, the increase in ALP was an isolated biochemical finding. It occurred while ALT levels significantly decreased and bilirubin levels remained unchanged. Furthermore, this effect was species-specific, as it was not observed in ICR mice. Most importantly, this enzymatic fluctuation was not supported by any histopathological evidence of liver or bile duct damage.

The observed reductions in markers like creatinine, ALT, BUN, and triglycerides are generally indicative of normal or improved physiological function rather than impairment. While ALP was elevated in female rats and BUN and cholesterol were elevated in female mice, these modest changes were not accompanied by any corresponding histopathological evidence of tissue damage, as will be discussed in Section 2.2.3. Instead of being indicators of toxicity, such slight variations without a pathogenic link are frequently seen as incidental or adaptive reactions [36].

#### 2.2.2. Hematology

Hematological analysis also revealed some modest alterations (Table 6). When female Wistar rats were treated with SL, their platelet counts and WBC significantly increased while their neutrophil numbers decreased. A significant increase in monocytes was observed in female ICR mice. Without apparent inflammatory lesions, these slight changes in leukocyte populations are likely a sign of the extract’s mild, benign immunomodulatory effect. This interpretation is consistent with previous reports on the biological activities of both lutein and sericin components [20,37]. Therefore, when considered in their entirety, the data from the acute toxicity study robustly demonstrate the safety of SL at high-dose administration.

#### 2.2.3. Histopathological Findings from Acute Oral Toxicity Study

Histopathological examination of all collected organs revealed no treatment-related microscopic lesions in any of the SL-treated animals. There was no indication of cellular degeneration, necrosis, or inflammation in any of the tissues, including the liver, kidneys, spleen, and thymus, and their cellular architecture was similar to that of the corresponding control groups.

Crucially, these findings provide a definitive context for interpreting the biochemical and hematological variations observed. The normal histology of the liver and kidneys confirms that the statistically significant elevations in ALP (in female rats) and BUN (in female mice) (Table 5) were indeed toxicologically irrelevant and did not represent organ injury. The absence of a pathological correlation serves as significant evidence indicating that these fluctuations were incidental rather than detrimental [36]. Similarly, the normal histology of the spleen and thymus corroborates the interpretation that the alteration in leukocyte counts (Table 6) was likely due to a non-adverse immunomodulatory response rather than a sign of systemic inflammation [38].

Therefore, the histopathological examination provides unequivocal evidence at the cellular level that a high, single dose of SL is well-tolerated and does not induce acute organ toxicity.

### 2.3. Chronic Oral Toxicity Study

#### 2.3.1. Body and Visceral Organ Weight

The six-month chronic study was designed to investigate the long-term safety and physiological effects of SL. Daily administration of SL at doses up to 50 mg/kg BW did not result in any mortality, adverse clinical signs, or significant changes in body weight gain in either male or female Wistar rats (Table 7). Gross and microscopic histopathological examinations revealed no treatment-related lesions in any of the organs evaluated. While a statistically significant increase in the relative kidney weight was observed in female rats at all doses, this was not accompanied by any changes in renal function markers such as BUN or creatinine (Table 8) or any abnormal kidney histology. Thus, rather than being an indication of nephrotoxicity, this is regarded as a non-adverse adaptive response, a behavior occasionally seen with drugs that undergo substantial metabolic processing [36,39,40].

#### 2.3.2. Clinical Chemistry

In addition to demonstrating its fundamental safety, the study showed that long-term SL administration caused an array of significant, benign physiological changes, underscoring its promise as a bioactive nutraceutical. The most consistent and potent of these was a significant lipid-lowering effect (Table 8). The SL consistently and effectively lowered serum triglycerides in both sexes. This suggests that the SL complex modulates lipid metabolism through pathways distinctly and potentially clinically relevant. In female rats, a clear dose-dependent reduction in serum triglycerides was observed at all three SL doses. A similar, significant reduction was seen in male rats at the 25 and 50 mg/kg BW doses. One well-established advantageous bioactivity of carotenoids, which are known to alter lipid metabolism pathways, is their hypotriglyceridemic effect [41].

This consistent efficacy stands in sharp contrast to the toxicity profile of marigold-derived lutein reported by Harikumar et al. (2008) [40]. In their 13-week sub-chronic assessment, the administration of non-esterified lutein shows no significant effect on lipid profiles, while lutein esters yielded inconsistent and non-dose-dependent fluctuations in lipid profiles. While they observed a significant decrease in triglycerides in male rats at a low dose of 4 mg/kg BW, this effect was lost at higher doses (40 and 400 mg/kg BW) and was entirely absent in female rats, leading the authors to conclude that the extract produced no biologically relevant changes in lipid metabolism. Lutein esterification has been investigated as a strategy to mitigate this limitation and improve bioavailability [42]; however, our findings showed uniform efficacy of SL in serum triglycerides lowering effect, likely attributable to its unique naturally occurring protein-bound form embedded in fat matrix, which may enhance the stability and absorbability [15], allowing SL to actively regulate lipid metabolism.

The clinical data compiled in a recent meta-analysis by Ghasemi et al. (2023) [43] is strikingly consistent with the strong and targeted effect on triglycerides, with a negligible effect on total cholesterol. Their analysis of human trials concluded that lutein and zeaxanthin supplementation significantly reduces triglyceride levels but has no significant effect on total cholesterol, LDL, or HDL levels. The possible mechanisms for this specific triglyceride-lowering action are multifaceted. Lutein and zeaxanthin are thought to exert their effects primarily by modulating the key transcription factors that govern lipid metabolism [44,45]. It is plausible that SL acts through the activation of the central metabolic regulator, AMP-activated protein kinase (AMPK), a pathway typically initiated by an increase in the intracellular AMP/ATP ratio [46]. Once activated, AMPK exerts a dual, coordinated effect on lipid metabolism. It primarily inhibits lipogenesis by markedly reducing the mRNA expression of the master transcription factor SREBP-1c and its essential downstream targets associated with fatty acid and triglyceride synthesis, such as acetyl-CoA carboxylase (ACC), fatty acid synthase (FAS), and glycerol-3-phosphate acyltransferase (GPAT) [45]. However, further in vitro or in vivo studies of these signaling pathways are necessary to investigate for definitive validation. Specifically, molecular validation would involve assessing the phosphorylation status of AMPK (p-AMPK/AMPK ratio) and its downstream effectors, such as ACC, via Western blot analysis in liver and adipose tissues. Additionally, qPCR could be utilized to evaluate the mRNA expression of lipogenic transcription factors, such as SREBP-1c, to confirm this metabolic shift. Second, by increasing the expression of carnitine palmitoyltransferase-1 (CPT-1), the rate-limiting enzyme that carries fatty acids into the mitochondria for β-oxidation, it concurrently encourages fatty acid oxidation [46]. This combined action of robustly inhibiting lipid synthesis while enhancing lipid breakdown possibly provides a molecular explanation for the net reduction in triglyceride accumulation observed in our study. This dual action simultaneously suppressing the creation of new fat while enhancing the burning of existing fat, provides a robust molecular explanation for the strong hypotriglyceridemic effect observed in our study.

This targeted effect on triglyceride metabolism distinguishes lutein and zeaxanthin from other well-known carotenoids. For instance, lycopene, the primary carotenoid in tomatoes, is most frequently associated with lowering cholesterol. Similar to statin medications, its suggested mode of action involves inhibiting HMG-CoA reductase, the rate-limiting enzyme in the cholesterol production pathway [47,48]. In contrast, carotenes like β-carotene and α-carotene primarily function as precursors to vitamin A (retinoic acid). Through the activation of retinoic acid receptors (RARs), which can affect the expression of a large number of genes, their effects on lipid metabolism often occur indirectly [49,50]. Therefore, while many carotenoids exhibit beneficial effects on lipid profiles, the specific molecular pathways they target appear to differ. The bioactivity of SL is attributed to the specific metabolic modulations of lutein and zeaxanthin, as evidenced by the strong and isolated reduction in triglycerides. This regulation is focused on the synthesis and oxidation of fatty acids, rather than the cholesterol synthesis pathway that is targeted by compounds such as lycopene.

The data presented in Table 8 for male Wistar rats revealed the significant reduction in total protein and total bilirubin at the 50 mg/kg BW dosage appears to be a non-adverse, adaptive response rather than an indication of hepatotoxicity. Although total protein levels decreased to 6.50 g/dL, the hepatic synthetic function remains intact, as evidenced by the stable albumin levels (3.40 g/dL), which did not differ significantly from the control [51]. Furthermore, while elevated bilirubin is a standard marker for liver dysfunction or hemolysis, a decrease in total bilirubin is clinically benign; this reduction may be attributed to the antioxidant properties of SL, which could lower oxidative stress and consequently reduce the physiological demand for bilirubin [52]. Collectively, the absence of elevated liver enzymes (AST and ALT) supports the conclusion that the liver’s structural and functional integrity was preserved despite these biochemical variations [53].

#### 2.3.3. Hematology

Hematological analysis also revealed some modest, sex-specific alterations that were not considered toxicologically significant (Table 9). In male rats, a slight but significant decrease in WBC count was observed at the higher doses, while hemoglobin levels increased at the 50 mg/kg BW dose. A distinct trend was observed in female rats, where the higher doses resulted in a drop in lymphocytes and an increase in neutrophils. In the absence of any inflammatory lesions in key immune organs like the spleen and thymus, these minor fluctuations are interpreted as a mild, non-adverse immunomodulatory response, consistent with previous reports on the biological activities of both lutein and its protein carrier [20,37]. Specifically, Promphet et al. (2014) [20] demonstrated that oral administration of SL extract (10–20 mg/kg) to BALB/c mice significantly enhanced both innate and adaptive immune functions, evidenced by a dose-dependent increase in Natural Killer (NK) cell activity and an upregulation of CD3+ and CD4+ lymphocyte subpopulations. This contrasts with an equivalent dose of marigold lutein, which proved largely ineffective. This immunostimulatory potential helps explain the shift in neutrophil counts observed in the female rats, suggesting an active innate immune engagement.

In addition to the lutein molecule itself, the co-extracted matrix and significant variations in its chemical form provide the mechanistic basis for this improved bioactivity. Commercial marigold lutein is typically present in a free or diesterified form, but the lutein in the silk extract is naturally found in its protein-bound form [11,12,54]. Free lutein from marigold exhibits significantly higher bioavailability than its counterpart esterified form, as the human body does not require enzymatic hydrolysis of ester bonds in the gut before absorption [55].

Second, and possibly more importantly, the method for extracting SL that Promphet et al. (2014) [20] describe produces a natural formulation that contains a considerable amount of co-extracted lipids, specifically unsaturated and saturated fatty acids such as oleic, palmitic, stearic, and linoleic acids, respectively. These lipids are crucial for the increased absorption of xanthophylls, such as lutein, since they help the intestines create micelles, which is a necessary step for the carotenoid to be dissolved and transported across the enterocyte wall [56]. In contrast, purified marigold extracts lack this intrinsic lipid matrix, making their absorption highly dependent on the fat content of the co-ingested meal. Therefore, the SL investigated in our study is not merely lutein, but a synergistic, lipid-based formulation. The combination of a natural lipid carrier and a proven chemical form found in lutein pigment in macula offers a convincing explanation for the stronger immunomodulatory response lutein produced from silk, which is in line with the hematological alterations seen in our investigation.

#### 2.3.4. Effects on Blood Pressure and Blood Glucose Level

**Figure 4:**
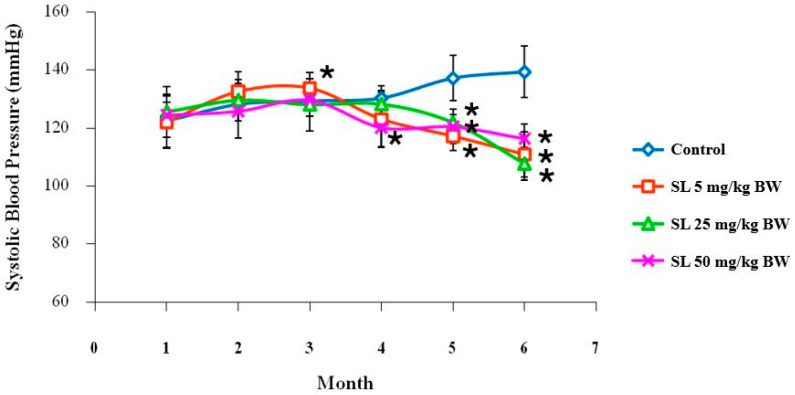
Systolic blood pressure of male Wistar rats in the control group compared with the group treated with various concentrations of SL extract for 6 months. Values are expressed as mean ± SD. * *p* < 0.05; Six rats per group.

**Figure 5:**
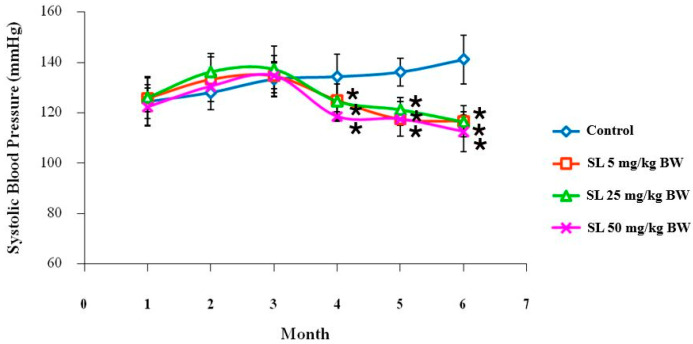
Systolic blood pressure of female Wistar rats in the control group compared with the group treated with various concentrations of SL extract for 6 months. Values are expressed as mean ± SD. * *p* < 0.05; Six rats per group.

The most striking finding from the in-life monitoring was a powerful and sustained hypotensive effect (Figure 4 and Figure 5). While a transient increase in SBP was noted in the 5 mg/kg BW male group at month 3, a consistent and powerful hypotensive effect emerged thereafter. From month 4 forward, SL treatment dramatically reduced the age-related, natural progression to age-related hypertension seen in the control group in both sexes [57,58,59]. The development of hypertension is closely intertwined with aging, driven by a combination of pathological factors described as the “Vascular Health Triad” [60], which encompasses chronic inflammation, oxidative stress, and vascular dysfunction [59]. At the conclusion of the study, the SBP in all groups treated with SL was sustained at a healthy baseline of approximately 120 mmHg, in sharp contrast to the control rats, whose SBP surpassed 140 mmHg. This potent vasoregulatory effect is consistent with previous studies demonstrating that lutein can improve endothelial function and nitric oxide bioavailability through the antioxidant properties of lutein [16,58].

The potent hypotensive effect of SL observed in this study, particularly the sustained maintenance of systolic blood pressure at approximately 120 mmHg from month 3 onwards, aligns with but distinctively extends current mechanistic understanding. Mechanistically, lutein is known to restore endothelial function by upregulating the bioavailability of the vasodilator NO while simultaneously downregulating the potent vasoconstrictor endothelin-1 (ET-1) [61,62]. The observed hypotensive impact of SL in this study appears to be consonant with the enhancement of NO bioavailability and the preservation of endothelial function, as previously reported in other animal models [16]. However, in the absence of direct molecular data in the current investigation, this mechanism remains a hypothesis to be validated. Future in vivo or ex vivo studies would be necessary to confirm this signaling pathway, potentially through the measurement of systemic markers of endothelial function, such as serum nitrate/nitrite levels and the expression of endothelial nitric oxide synthase (eNOS), or via aortic ring assays to evaluate direct vasorelaxation responses. Additionally, lutein may act as a vascular antioxidant by inhibiting NADPH oxidase activity, thereby reducing the reactive oxygen species (ROS) that degrade NO [63]. However, a critical distinction exists between the present findings and previous reports. Sung et al. (2013) [16] demonstrated that commercial lutein (2 mg/kg BW) could reverse hypertension in an Nω-nitro-L-arginine methyl ester (L-NAME) model within just 3 weeks. In contrast, our study validates the efficacy of SL in a chronic, age-related model, where vascular dysfunction arises from gradual structural and metabolic decline rather than immediate chemical toxicity. The emergence of the hypotensive effect at Month 3 in our study suggests that SL effectively counters these chronic, systemic age-related changes, offering a sustained defense against vascular aging that goes beyond the rapid reversal seen in acute chemical models.

In male rats, the 25 and 50 mg/kg BW doses induced significant reductions in blood glucose at several time points (Table 10), with the most consistent effect seen at the highest dose in the final months. This effect was absent in female rats, suggesting a potential interaction between SL and sex-specific hormonal regulation of glucose metabolism, a phenomenon that warrants further investigation [64]. While the reduction in blood glucose in the high-dose male groups was statistically significant, the values remained well within the normal physiological reference range for Wistar rats [65]. This indicates that SL functions as a metabolic regulator rather than a hypoglycemic agent, improving glucose handling without inducing dangerous hypoglycemia. The observed sex-specific efficacy is likely attributable to the inherent protective role of estrogen in female rats. Estrogen is known to enhance insulin sensitivity and protect pancreatic β-cell function against oxidative stress, thereby maintaining tighter glucose homeostasis naturally [66,67]. In contrast, male rats are more susceptible to age-related insulin resistance and visceral adiposity as they mature [68]. This indicates that SL functions as a metabolic regulator rather than a potent hypoglycemic agent, improving glucose handling without inducing dangerous hypoglycemia. The observed hypoglycemic activity is likely driven by the potent antioxidant capacity of lutein, which protects pancreatic β-cells from oxidative stress-induced dysfunction, thereby preserving insulin secretion capacity [69]. Furthermore, lutein has been shown to activate AMP-activated protein kinase (AMPK), a central regulator of energy homeostasis that enhances glucose uptake in peripheral tissues [70].

Ultimately, SL is ideally suited for integration into the human diet as a high-potency nutraceutical or a functional food additive. Its high lipid content (94.86%) enables it to function as a “self-delivering” system, facilitating micellarization and intestinal absorption without the strict necessity of a high-fat meal. This natural lipid-sericin matrix also provides a protective environment that enhances the stability and bioavailability of the carotenoid compared to conventional free lutein [20]. Potential applications include formulation into soft gel capsules or incorporation into various food matrices, providing a stable delivery format that ensures consistent physiological benefits regardless of the user’s specific dietary fat intake.

To contextualize the findings of this study within the framework of human clinical relevance, the rodent dosages were translated to the Human Equivalent Dose (HED) using the Body Surface Area (BSA) normalization method as recommended by Reagan-Shaw et al. (2008) [71]. Based on a conversion factor of 0.162 (derived from a rat $K_{m}$ of 6 and a human $K_{m}$ of 37), the daily oral dosages of 5, 25, and 50 mg/kg BW administered to rats translate to HEDs of 0.81, 4.05, and 8.1 mg/kg, respectively. For a standard 60 kg adult, these dosages correspond to a daily intake of 48.6 mg, 243 mg, and 486 mg of lutein content contained within 1.39 g, 6.94 g, and 13.9 g of the crude SL extract, respectively.

Given that the SL extract contains 3.5–5.4% *w*/*w* lutein, these ranges are highly relevant to human health applications, as they align with the upper therapeutic levels utilized in major clinical trials. For instance, the AREDS2 study utilized 10 mg of lutein daily [72]. This demonstrates that the cardiovascular benefits observed in our study occur at a dosage that is easily achievable in humans (approx. 1.39–13.9 mg) of the crude SL extract. These calculations reinforce the practical applicability of SL as a concentrated, multipurpose nutraceutical for age-related cardiovascular conditions.

## 3. Materials and Methods

### 3.1. Test Materials

The novel SL extract was derived from yellow silk cocoons (*Bombyx mori*, Nangnoi variety) obtained from Sericulture farms and Queen Sirikit Sericulture Centers in the Thai provinces of Nakhon Ratchasima, as detailed in Manupa et al. (2023) [15] and Sutheerawattananonda et al. (2015) [73]. SL extract was stored at –20 °C in the dark until use. Butylated hydroxyanisole (BHA), tocopherol, and analytical standards of lutein (X6250), kaempherol (60010), and quercetin (Q4951) were purchased from Sigma-Aldrich Co., Ltd. (MO, USA). Carlo Erba Reagents supplied methanol, acetonitrile, ethanol, ethyl acetate, Folin–Ciocalteu reagent, and n-hexane of high-performance liquid chromatography (HPLC) grade (Arese, Italy).

### 3.2. SL Extraction

Approximately 1 g of de-pupated, Nangnoi variety of Thai yellow silk cocoons were soaked in distilled water at a 1:30 (*w*/*v*) ratio and subsequently degummed by autoclaving at 121 °C for 15 min. The resulting sericin-rich aqueous solution was separated from the degummed cocoon fibers by filtration. Following that, lutein was extracted from the fibrous components and the aqueous solution in three consecutive extractions using 30 mL of a solvent mixture consisting of hexane, ethyl alcohol, and ethyl acetate (3:2:1, *v*/*v*/*v*), supplemented with 0.1% butylated hydroxyanisole (BHA, *w*/*v*). The pooled organic extracts were then subjected to liquid–liquid partitioning by adding 100 mL of 10% (*w*/*v*) NaCl solution to facilitate phase separation. This partitioning step was repeated with fresh organic solvent until the aqueous phase became colorless. The final organic phase was collected and concentrated to dryness under reduced pressure using a rotary evaporator (BUCHI, Flawil, Switzerland) at 35 °C. Immediately after, the dried extract was purged with nitrogen gas, placed in amber vials with screw caps, and kept at −80 °C until its further analysis.

### 3.3. Characterization and Analysis of SL Extract

#### 3.3.1. Lutein Quantification by HPLC

The lutein content in the SL extract was quantified by HPLC analysis, performed on an Agilent HP 1120 Compact LC system equipped with a photodiode array detector, following a previously described method by Manupa et al. (2023) [15]. Chromatographic separation was achieved on a LiChrospher^®^ 100RP-18C (5 µm particle size, 250 × 4.6 mm internal diameter) column (Merck, Darmstadt, Germany) maintained at 20 °C with the mobile phase consisting of a mixture of solvent A (acetonitrile: methanol, 9:1 *v*/*v*) and solvent B (ethyl acetate) at a flow rate of 0.5 mL/min. In order to prepare the samples, the extract was dissolved in 100% ethanol and then filtered through a 0.45 µm syringe filter into a 5.00 mL amber vial. A gradient elution protocol was employed for the mobile phase—20% B for 15 min, 20–50% B for 15–20 min, 50–20% B for 20–25 min, and maintained at 20% B for 25–40 min. The injection volume was 10.0 µL, and lutein was detected at a wavelength of 445 nm. Data acquisition and analysis were conducted using Plumbagin software Version B.01.03 SP4 (Agilent Technologies, Waldbronn, Germany).

#### 3.3.2. Safety and Quality Assessment

For comprehensive safety and quality assessment, the SL extract was analyzed by a certified third-party laboratory (Central Laboratory Co., Ltd., Khon Kaen, Thailand). Trace metal analysis (Pb, Cd, Hg, As, Zn, and Ni) was performed by an ISO/IEC 17025 [74] accredited facility using Inductively Coupled Plasma Mass Spectrometry (ICP-MS) following microwave-assisted acid digestion (EPA Method 3052) [75]. Analytical validity was established through calibration curves with $R^{2}>0.999$ and a specific Limit of Detection (LOD).

Microbiological safety was evaluated for Coliforms and *E. coli* (FDA BAM Online, 2017) [76], *Listeria monocytogenes* (ISO 11290-1:2017) [77] *Salmonella* spp. (ISO 6579-1:2017) [78], *Staphylococcus aureus* (FDA BAM Online, 2016) [79], total plate count, and yeasts and molds (FDA BAM Online, 2001) [80]. Pesticide residue analysis was performed using a QuEChERS-based method (EN 15662:2018) [81], with carbamates analyzed by LC-MS, and organochlorine, organophosphate, and pyrethroid groups assessed by GC/ECD and GC/FPD, respectively.

The compositional profile of the Silk Lutein (SL) extract was determined using a multi-analytical approach to characterize the lipid matrix and ensure the reliability of the bioactive quantification. Total lipid content was determined gravimetrically by evaporating the hexane phase to dryness and weighing the resulting residue. To provide a detailed profile of the lipophilic components, fatty acid compositions (C4–C24) were analyzed via gas chromatography utilizing a Hewlett Packard GC system HP6890 A, equipped with a 100 m × 0.25 mm fused silica capillary column (SP2560, Supelco Inc, Bellefonte, PA, USA). The injector and detector temperatures were set at 250 °C. The oven temperature program was as follows: 4 °C for 4 min, increased at 13 °C/min to 175 °C (held for 7 min), and finally increased at 4 °C/min to 215 °C (held for 17 min) [20]. The content of long-chain fatty acids (C > 24) and waxes was subsequently estimated through mass balance, calculating the difference between the total fat and the GC-quantified C4–C24 fraction, and further confirmed by gravimetric analysis on a dry weight basis.

To ensure analytical reliability and eliminate potential interference from the ~94.86% fat/wax matrix, lutein was quantified using reversed-phase high-performance liquid chromatography (RP-HPLC) on an Agilent HP 1100 series system. The high specificity of the RP-HPLC method allowed for the distinct chromatographic separation of the lutein peak from the complex lipid and wax components. The identity of lutein was further confirmed through liquid chromatography-mass spectrometry (LC-MS) utilizing an Ultraflex III TOF/TOF (Bruker Daltonik, GmbH, Bremen, Germany) [20]. LC-MS analysis also confirmed that kaempherol and quercetin were not detected in the SL extract.

As part of the routine quality control and to verify the presence of the wax matrix, the extract was analyzed using a UV/Vis spectrophotometer (Libra S22, Biochrom Ltd., UK) across a scanning range of 190 to 1000 nm, with results compared against an authentic lutein standard (Sigma-Aldrich, Darmstadt, Germany). Finally, the moisture content was assessed using the AOAC method 926.12 [82] by drying samples at 65 °C under a pressure of 100 mmHg until a constant weight was achieved.

#### 3.3.3. Preparation for Animal Administration

Stock solutions of SL were prepared for animal administration based on the lutein concentration determined by HPLC. The lutein extract was solubilized in dimethyl sulfoxide (DMSO) and then dissolved in 1% Tween 80 in phosphate-buffered saline (PBS) at pH 7.4 to achieve the desired concentrations for oral gavage. This working lutein solution was made once a week and kept in sealed containers under nitrogen gas at −20 °C in the dark.

### 3.4. Animals

Twelve female Wistar rats (8 weeks old) and twelve female ICR mice (20–30 g) were obtained from the National Bureau of Laboratory Animals, Mahidol University (Salaya, Nakhon Pathom, Thailand) for the acute toxicity tests. For the chronic toxicity testing, 24 male and 24 female Wistar rats were used. The total number of animals used for both studies was 72. The animals were housed in a room with a 12:12 h light-dark cycle at a temperature of 25 ± 2 °C and provided with standard rodent feed (C.P. Company, Bangkok, Thailand) and ad libitum access to reverse osmosis (RO) water throughout the study. The animal research was conducted in accordance with the Code of Ethics for the Use of Animals for Scientific Work, National Research Council of Thailand and the protocol was approved by the animal ethics committee of the Faculty of Medicine, Chiang Mai University (Protocol No. 7/2555). To ensure transparent and reproducible reporting, the study followed the ARRIVE Essential 10 guidelines, as detailed in the Compliance Questionnaire (File S1).

Following each feeding trial, the animals were euthanized by an intraperitoneal injection of a lethal dose of 50 mg/kg BW of thiopental sodium (THIOPENTAL, Biopharma, Bangkok, Thailand). Blood was collected immediately by cardiac puncture. Subsequently, the liver, kidney, adrenal gland, pancreas, heart, lung, spleen, prostate gland, seminal vesicle, epididymis, testis, and ovary were aseptically removed, and their weights were recorded [57].

### 3.5. Study Design

#### 3.5.1. Acute Oral Toxicity Study

The acute oral toxicity study was conducted for the novel SL extract only, as a limit test, in accordance with OECD Test Guideline 423 [29]. Although standard protocols mention both sexes, females are commonly selected for acute toxicity testing because they tend to be more sensitive to chemical exposure than males [83,84]. In accordance with this rationale and the 3Rs principle of reducing animal numbers [85] the current study was performed on female ICR mice and female Wistar rats to establish the safety profile of the novel SL extract. The animals were divided into 2 groups (*n* = 6 per species per group: Group 1 (Control): Received 1 mL of distilled water, Group 2 (SL-treated): Received a single oral gavage dose of SL at 2000 mg/kg BW. Following administration, all animals were closely monitored for clinical signs of toxicity (including mortality, gastrointestinal discomfort, ataxia, emesis, and tremors) continuously for the first four hours and then daily for a 14-day observation period, during which they had unrestricted access to food and water [57]. Body weights were recorded prior to dosing (Day 0) and at the conclusion of the study (Day 14). At the end of the observation period, all surviving animals were euthanized, and blood and internal organs were collected for subsequent histological, hematological, and serum biochemical examinations.

#### 3.5.2. Chronic Oral Toxicity Study

For the chronic toxicity study, male and female Wistar rats were randomly divided into four groups: (*n* = 6 per sex per group): Group 1 (Control): Received 1 mL of distilled water, Group 2–4 (SL-treated): Received SL at dosages of 5, 25, or 50 mg/kg BW/day. The study was conducted for a duration of 6 months.

### 3.6. In-Life Observations and Measurements During Chronic Oral Toxicity Study

Throughout the study, animals were monitored for clinical signs of toxicity, and body weights were recorded at the beginning and conclusion of this investigation. Blood glucose levels were assessed monthly using blood samples from the tail vein with Accu-Chek instant glucose test strips (ACCUCHEK, Roche Laboratories, Pharma, Mannheim, Germany). Systolic blood pressure (SBP) was measured using a non-invasive tail-cuff method. Prior to SBP measurement, rats were acclimated in a heating box (PMB-1030 Preheat & Measuring Box, MUROMACHI KIKAI Co., Ltd., Tokyo, Japan) at 37 °C for 15 min to enhance the detection of the tail artery pulse. The final SBP was determined as the mean of three consecutive readings, provided that the readings did not vary by more than 10 mmHg, as described by Tocharus et al. (2024) [57].

### 3.7. Terminal Procedures and Analysis

At the conclusion of the 6-month study, all animals were euthanized for blood and internal organs collection. Subsequently, internal organ weights were recorded. Blood samples were subjected to clinical chemistry and hematological analysis as detailed in the subsequent sections. All collected organs were preserved in 10% neutral buffered formalin for histopathological examination.

#### 3.7.1. Clinical Chemistry

Serum samples were analyzed for a panel of parameters including kidney function markers (blood urea nitrogen (BUN), creatinine); liver function markers (aspartate aminotransferase (AST), alanine aminotransferase (ALT), alkaline phosphatase (ALP), total bilirubin, direct bilirubin, total protein, albumin); and a lipid profile (cholesterol, high-density lipoprotein (HDL), low-density lipoprotein (LDL), triglycerides). Uric acid was also measured [86].

#### 3.7.2. Hematology

EDTA-anticoagulated whole blood was analyzed for a complete blood count (CBC) with differential. The evaluated parameters comprised hemoglobin (Hb), hematocrit (Hct), red blood cell (RBC) count, platelet count, and RBC indices, specifically mean corpuscular volume (MCV), mean corpuscular hemoglobin (MCH), and mean corpuscular hemoglobin concentration (MCHC). The white blood cell (WBC) differential count included neutrophils, lymphocytes, monocytes, eosinophils, and basophils [87].

#### 3.7.3. Histopathological Examination

Organs such as the heart, lungs, liver, kidneys, adrenal glands, prostate glands, testes, and ovaries were gathered, weighed, and preserved in 10% formalin for histological examination. The fixed tissues underwent standard processing and were sectioned at a thickness of less than 3 μm. Following that, the sections were stained for microscopic analysis using hematoxylin and eosin (H&E) [88]. All tissues were evaluated by a pathologist based on the Standardized System of Nomenclature and Diagnostic Criteria (SSNDC) and the Best Practices Guideline for Toxicologic Histopathology [36,89].

### 3.8. Statistical Analysis

All experimental data are expressed as the mean ± standard deviation (SD). Statistical comparisons were tailored to the specific design of each study phase. For the acute toxicity study, which involved a direct comparison between two independent cohorts (Control vs. 2000 mg/kg BW), an independent-samples *t*-test was employed to assess differences in physiological and biochemical parameters. For the chronic toxicity study, which evaluated longitudinal data across multiple treatment levels (5, 25, and 50 mg/kg BW) and vehicle control, one-way analysis of variance (ANOVA) was utilized to determine the variance between groups at each specific time point. In instances where ANOVA yielded significant results, Fisher’s Least Significant Difference (LSD) post hoc test was performed for pairwise comparisons to identify specific differences between the treatment groups and the vehicle control. Statistical significance for all tests was defined a priori at the p<0.05 level, and this threshold was consistently applied and clearly indicated across all tables and figures to ensure clarity in the interpretation of the extensive datasets. All statistical computations were performed using SPSS software (Version 16.0, SPSS Inc., Chicago, IL, USA).

## 4. Conclusions

This study provides the first comprehensive toxicological evaluation of SL, a novel, protein-bound lutein complex from yellow silk *Bombyx mori* cocoons. The findings robustly demonstrate the safety of SL for long-term oral consumption, with an acute LD_50_ greater than 2000 mg/kg BW and no evidence of cumulative toxicity following six months of daily administration. The modest physiological changes that were seen had no negative effects and no pathological correlations. This demonstrates the fundamental safety of SL as a possible constituent in nutraceuticals. Beyond establishing its safety, this study revealed that SL is a multi-functional bioactive agent. The most significant findings were the potent and sustained hypotensive and triglyceride-lowering effects observed in the chronic study. The results provide the first in vivo proof that this new protein-bound form of lutein, when taken orally over a lengthy period of time, provides significant cardiovascular benefits in addition to its well-established roles in immunological and ocular health. While the precise mechanisms were not the focus of this safety study, the observed vasoregulatory effects are consistent with the known antioxidant properties of lutein. Ultimately, SL is more than just a stable and safe substitute for traditional lutein sources; it is a multipurpose component that simultaneously promotes cardiovascular and optical health. Furthermore, the production of SL extract represents a cost-effective and sustainable advancement in carotenoid sourcing. By repurposing underutilized yellow silk cocoons from the textile industry, SL facilitates resource valorization within a circular bioeconomy, which provides a significant economic advantage over traditional lutein sources, offering a high-value nutraceutical constituent with a reduced environmental footprint. The data presented herein establish a robust basis for its advancement as a next-generation nutraceutical aimed at holistic health and healthy aging. Further mechanistic studies are required to elucidate the specific pathways through which this unique lutein complex exerts its notable physiological effects.

## Figures and Tables

**Table 1:** Specifications of SL from yellow silk cocoons.

Items	Specifications	
Source	Silk cocoon	
Molecular Formula	C_40_H_52_O_2_	
Molecular Weight	568.88	
Shelf Life	Nine months in dark packing at −20 °C [15]	
Total lutein in silk extract	3.50–5.40%	
% Moisture	approx. 1 ± 0.13%	
**Fat:**	94.86%	
- Free fatty acid (C4–C24)	9.84%	
- Flavonoid (Kaempferol, Quercetin: C15)	Not Detected	
- Free fatty acid (C > 24)	84.99%	
- Lutein (C40)	4.12–6.35%	
- Wax	79.59–81.49%	
**Other (Large Molecular Weight Wax)**	approx. 4.14%	
**Trace Metals:**	**(** **mg/kg crude extract** **)**	**Limits of Detection (LOD) (mg/kg)**
- Pb max	0.740	0.025
- Cd max	0.068	0.025
- Hg max	0.803	0.025
- As max	0.040	0.025
- Zn max	16.505	0.250
- Ni max	0.942	0.250
**Microbiology:**		
- Standard plate counts	<1.0 cfu/g	
- Yeast & Molds	<10 cfu/g	
- *E. coli*	<3.0 MPN/g	
- Salmonella spp.	Not Detected	
**Pesticide Residue:**		
- Organochlorine Group	Not Detected	
- Organophosphate Group	Not Detected	
- Pyrethroid Group	Not Detected	
- Carbamate Group	Not Detected	

**Table 2:** Comparison of the maximum tolerable daily intake (TDI) of trace ions with the trace metal levels resulting from the daily consumption of 6 mg of SL extract.

Trace Metal	TDI	Heavy Metal Contamination (in Lutein/6 mg)	Reference
Pb	15 µg/day for greater than 7 years	0.081–0.126 µg (0.54–0.84% of TDI)	Wong et al. (2022) [24]
	25 µg/day for pregnant women	(0.32–0.50% of TDI)	
	75 µg/day for other adults	(0.11–0.17% of TDI)	
Cd	6 µg/day	0.007–0.012 µg (0.12–0.20% of TDI)	Filippini et al. (2019) [25]
Hg	2 µg/day	0.088–0.137 µg (4.40–6.85% of TDI)	(USEPA, 2024) [23]
As	10 µg/day	0.004–0.007 µg (0.04–0.07% of TDI)	(WHO, 2017) [26]
Zn	15,000 µg/day for men	1.816–2.806 µg (0.01–0.02% of TDI)	Cabrera Á (2015) [27]
	12,000 µg/day for women	(0.01–0.02% of TDI)	
Ni	5 µg/kg BW/day	0.103–0.160 µg (2.06–3.20% of TDI)	Manfredi et al. (2025) [28]

**Table 3:** Analytical results of standard lutein and SL determined by LC-MS.

**Standard Lutein**	***m*** **/*z***	549.6	550.5	551.7	567.5	568.6	569.7	-
	**Intensity**	13,255	31,272	22,668	34,628	65,124	30,997	-
**SL**	***m*** **/*z***	549.6	550.8	551.6	567.7	568.7	569.7	598.5
	**Intensity**	16,006	13,675	1944	12,430	5173	637.9	10,376

**Table 4:** Comparison of initial and final body weights, as well as internal organ weights, of female Wistar rats and ICR mice in both the control groups with those given SL at a dose of 2000 mg/kg BW for 14 days.

Parameter	Female Wistar Rats		Female ICR Mice	
	Control	SL 2000 mg/kg BW	Control	SL 2000 mg/kg BW
Initial body weight (g)	198.33 ± 13.29	201.67 ± 9.83	22.83 ± 0.75	21.33 ± 1.51
Final body weight (g)	235.00 ± 21.68	245.00 ± 17.61	29.67 ± 0.82	29.50 ± 1.38
Liver (g)	7.40 ± 0.87	8.27 ± 1.65	1.46 ± 0.05	1.42 ± 0.04
Kidney (g)	1.44 ± 0.13	1.51 ± 0.13	0.37 ± 0.02	0.37 ± 0.02
Adrenal gland (g)	0.06 ± 0.01	0.06 ± 0.01	0.01 ± 0.00	0.01 ± 0.00
Pancreas (g)	0.63 ± 0.06	0.67 ± 0.06	0.20 ± 0.03	0.16 ± 0.03

Values are expressed as mean ± SD.

**Table 5:** Comparison of the clinical blood chemistry of female Wistar rats and ICR mice in both the control groups with those given SL at a dose of 2000 mg/kg BW for 14 days.

Parameter	Female Wistar Rats		Female ICR Mice	
	Control	SL 2000 mg/kg BW	Control	SL 2000 mg/kg BW
Glucose (mg/dL)	109.50 ± 4.76	107.17 ± 5.31	107.33 ± 3.56	104.17 ± 5.46
BUN (mg/dL)	35.92 ± 5.41	34.15 ± 3.14	22.38 ± 1.70	25.45 ± 1.04 *
Creatinine (mg/dL)	0.69 ± 0.04	0.62 ± 0.05 *	0.35 ± 0.04	0.34 ± 0.04
Uric acid (mg/dL)	1.85 ± 0.83	1.41 ± 0.48	3.71 ± 0.12	3.67 ± 0.20
Cholesterol (mg/dL)	58.33 ± 6.56	61.83 ± 3.49	85.50 ± 7.15	100.17 ± 5.12 *
HDL (mg/dL)	24.90 ± 3.30	26.52 ± 1.25	-	-
LDL (mg/dL)	71.10 ± 9.10	77.62 ± 4.65	-	-
Triglyceride (mg/dL)	60.67 ± 12.86	53.67 ± 12.32	-	-
Total protein (g/dL)	5.51 ± 0.17	5.60 ± 0.46	-	-
Albumin (g/dL)	3.27 ± 0.51	3.37 ± 0.06	-	-
Total bilirubin (mg/dL)	0.36 ± 0.06	0.39 ± 0.08	-	-
Direct bilirubin (mg/dL)	0.27 ± 0.10	0.20 ± 0.11	-	-
AST/SGOT (U/L)	111.00 ± 11.56	124.00 ± 17.20	97.83 ± 7.19	94.17 ± 9.20
ALT/SGPT (U/L)	43.00 ± 2.00	33.50 ± 1.87 *	21.67 ± 1.86	18.17 ± 2.14 *
ALP (U/L)	85.50 ± 16.60	114.67 ± 25.56 *	-	-

Values are expressed as mean ± SD * *p* < 0.05; Six rats per group.

**Table 6:** Comparison of the hematological data of female Wistar rats and ICR mice in both the control groups with those given SL at a dose of 2000 mg/kg BW for 14 days.

Parameter	Female Wistar Rats		Female ICR Mice	
	Control	SL 2000 mg/kg BW	Control	SL 2000 mg/kg BW
Hemoglobin (g/dL)	13.43 ± 0.21	13.13 ± 0.55	12.57 ± 0.26	12.83 ± 0.42
Hematocrit (%)	41.33 ± 0.52	38.00 ± 4.00	40.67 ± 1.03	40.83 ± 1.17
WBC (×10^3^ cell/uL)	1.80 ± 0.26	2.42 ± 0.56 *	5.67 ± 0.44	5.50 ± 0.97
Neutrophil (%)	10.17 ± 2.32	6.50 ± 1.64 *	8.17 ± 0.75	8.17 ± 1.47
Lymphocyte (%)	78.83 ± 2.93	81.17 ± 1.17	80.50 ± 1.52	79.17 ± 1.47
Monocyte (%)	10.33 ± 2.58	11.50 ± 0.55	10.67 ± 0.82	11.83 ± 0.98 *
Eosinophil (%)	0.67 ± 0.82	0.83 ± 0.98	0.50 ± 0.55	0.50 ± 0.55
Basophil (%)	0.00 ± 0.00	0.00 ± 0.00	0.17 ± 0.41	0.33 ± 0.52
Atypical lymphocyte (%)	0.00 ± 0.00	0.00 ± 0.00	0.00 ± 0.00	0.00 ± 0.00
Band form (%)	0.00± 0.00	0.00± 0.00	0.00± 0.00	0.00± 0.00
Platelet smear (%)	0.00± 0.00	0.00± 0.00	-	-
Platelet count (×10^3^ cell/uL)	530.33 ± 26.11	646.83 ± 51.83 *	742.67 ± 100.47	829.83 ± 42.72
MCV (fL)	48.17 ± 1.17	49.50 ± 1.52	46.17 ± 0.75	46.67 ± 0.52
MCH (pg)	15.83 ± 0.41	16.00 ± 0.89	14.67 ± 0.52	15.00 ± 0.00
MCHC (%)	32.83 ± 0.41	32.67 ± 0.52	31.33 ± 0.52	31.50 ± 0.55
RBC morphology	Normal	Normal	Normal	Normal

Values are expressed as mean ± SD * *p* < 0.05; Six rats per group.

**Table 7:** Body and internal weight of male and female Wistar rats in the control group compared with the group receiving SL extract at various concentrations for 6 months.

Parameter	Male Wistar Rats				Female Wistar Rats			
	Control	SL (mg/kg BW)			Control	SL (mg/kg BW)		
		5	25	50		5	25	50
Initial body weight (g)	232.17 ± 7.03	228.17 ± 3.49	228.00 ± 5.48	232.67 ± 5.32	202.83 ± 6.24	206.33 ± 8.55	211.00 ± 8.00	203.17 ± 7.68
Final body weight (g)	525.58 ± 47.56	527.57 ± 49.15	523.97 ± 35.85	525.76 ± 26.91	269.20 ± 11.56	279.97 ± 14.00	276.07 ± 20.87	267.60 ± 14.27
Heart (g)	1.23 ± 0.11	1.17 ± 0.14	1.31 ± 0.14	1.16 ± 0.08	0.78 ± 0.06	0.84 ± 0.03	0.83 ± 0.04	0.83 ± 0.07
Lung (g)	1.46 ± 0.16	1.38 ± 0.21	1.43 ± 0.17	1.42 ± 0.12	0.96 ± 0.06	1.02 ± 0.10	1.12 ± 0.25	1.04 ± 0.07
Liver (g)	14.52 ± 1.98	13.85 ± 1.43	13.59 ± 0.85	13.57 ± 0.22	7.24 ± 0.77	6.94 ± 0.55	6.84 ± 0.40	6.88 ± 0.67
Kidney (g)	2.24 ± 0.13	2.20 ± 0.22	2.23 ± 0.16	2.14 ± 0.06	1.35 ± 0.06	1.46 ± 0.09 *	1.53 ± 0.07 *	1.56 ± 0.11 *
Adrenal gland (g)	0.06 ± 0.02	0.07 ± 0.01	0.07 ± 0.00	0.07 ± 0.02	0.07 ± 0.01	0.06 ± 0.01	0.07 ± 0.01	0.07 ± 0.02
Pancreas (g)	0.83 ± 0.15	0.82 ± 0.14	0.96 ± 0.09	0.77 ± 0.28	0.65 ± 0.12	0.64 ± 0.12	0.67 ± 0.12	0.56 ± 0.08
Spleen (g)	0.72 ± 0.11	0.74 ± 0.07	0.82 ± 0.08	0.74 ± 0.07	0.57 ± 0.06	0.57 ± 0.05	0.65 ± 0.10	0.55 ± 0.08
Prostate gland (g)	0.41 ± 0.09	0.32 ± 0.14	0.34 ± 0.10	0.32 ± 0.13	-	-	-	-
Seminal vesicle (g)	1.20 ± 0.25	1.33 ± 0.23	1.10 ± 0.19	1.19 ± 0.23	-	-	-	-
Epididymis (g)	1.43 ± 0.31	1.35 ± 0.16	1.38 ± 0.10	1.40 ± 0.18	-	-	-	-
Testis (g)	3.82 ± 0.33	3.60 ± 0.18	3.79 ± 0.11	3.80 ± 0.35	-	-	-	-
Ovary (g)	-	-	-	-	0.11 ± 0.04	0.16 ± 0.14	0.11 ± 0.03	0.09 ± 0.04

Values are expressed as mean ± SD. * *p* < 0.05; Six rats per group.

**Table 8:** Clinical chemistry values of male and female Wistar rats in control group compared with the group receiving SL extract at various concentrations for 6 months.

Parameter	Male Wistar Rats				Female Wistar Rats			
	Control	SL (mg/kg BW)			Control	SL (mg/kg BW)		
		5	25	50		5	25	50
BUN (mg/dL)	28.50 ± 2.18	29.57 ± 2.28	29.97 ± 4.90	25.43 ± 2.81	34.33 ± 4.89	32.15 ± 4.82	35.95 ± 6.80	35.97 ± 8.75
Creatinine (mg/dL)	0.80 ± 0.12	0.73 ± 0.16	0.72 ± 0.04	0.73 ± 0.15	0.88 ± 0.05	0.83 ± 0.07	0.81 ± 0.24	0.74 ± 0.22
Uric acid (mg/dL)	1.81 ± 0.15	1.79 ± 0.07	1.77 ± 0.24	1.79 ± 0.14	2.22 ± 0.34	2.22 ± 0.37	2.28 ± 0.38	2.28 ± 0.33
Cholesterol (mg/dL)	52.00 ± 7.35	49.00 ± 7.67	42.50 ± 9.44	46.83 ± 7.55	32.33 ± 1.63	37.50 ± 3.39	35.67 ± 6.41	35.33 ± 6.77
HDL (mg/dL)	43.12 ± 2.73	42.42 ± 5.59	39.28 ± 5.82	38.00 ± 4.08	32.72 ± 2.96	35.30 ± 2.96	34.20 ± 4.90	36.40 ± 7.34
Triglyceride (mg/dL)	85.00 ± 9.76	79.67 ± 11.62	59.33 ± 17.65 *	59.83 ± 15.45 *	71.50 ± 9.91	58.50 ± 7.64 *	52.00 ± 7.07 *	45.33 ± 5.20 *
Total protein (g/dL)	6.77 ± 0.23	6.71 ± 0.14	6.70 ± 0.25	6.50 ± 0.18 *	6.40 ± 0.35	6.64 ± 0.30	6.70 ± 0.16	6.62 ± 0.36
Albumin (g/dL)	3.33 ± 0.38	3.54 ± 0.21	3.44 ± 0.27	3.40 ± 0.26	3.75 ± 0.30	3.53 ± 0.13	3.55 ± 0.30	3.55 ± 0.16
Total bilirubin (mg/dL)	0.64 ± 0.15	0.47 ± 0.17	0.48 ± 0.16	0.41 ± 0.15 *	0.48 ± 0.06	0.62 ± 0.04	0.53 ± 0.19	0.56 ± 0.11
Direct bilirubin (mg/dL)	0.13 ± 0.03	0.08 ± 0.06	0.11 ± 0.07	0.11 ± 0.03	0.11 ± 0.04	0.16 ± 0.06	0.13 ± 0.03	0.15 ± 0.03
AST/SGOT (U/L)	96.67 ± 7.76	94.00 ± 5.62	95.00 ± 8.12	99.50 ± 6.89	106.00 ± 13.90	113.50 ± 21.34	121.83 ± 13.32	115.83 ± 13.50
ALT/SGPT (U/L)	38.00 ± 7.38	34.33 ± 7.81	37.17 ± 6.97	38.00 ± 5.59	32.33 ± 4.03	35.83 ± 4.22	34.83 ± 2.23	34.67 ± 3.08
ALP (U/L)	117.17 ± 10.63	110.50 ± 22.42	103.50 ± 18.96	107.83 ± 25.00	88.67 ± 3.83	83.00 ± 16.88	88.83 ± 15.32	82.50 ± 9.52

Values are expressed as mean ± SD. * *p* < 0.05; Six rats per group.

**Table 9:** Hematological values of male and female Wistar rats in the control group compared with the group receiving SL extract at various concentrations for 6 months.

Parameter	Male Wistar Rats				Female Wistar Rats			
	Control	SL (mg/kg BW)			Control	SL (mg/kg BW)		
		5	25	50		5	25	50
Hemoglobin (g/dL)	14.65 ± 0.52	15.18 ± 0.70	15.25 ± 0.32	15.47 ± 0.89 *	14.57 ± 0.60	14.55 ± 1.22	14.58 ± 0.49	14.02 ± 0.29
Hematocrit (%)	44.00 ± 1.67	45.50 ± 2.43	45.83 ± 1.47	45.83 ± 2.79	42.17 ± 2.04	41.17 ± 1.94	42.33 ± 1.21	40.83 ± 1.33
WBC (×10^3^ cell/uL)	3.30 ± 0.44	3.62 ± 0.33	2.40 ± 0.58 *	2.58 ± 0.58 *	2.02 ± 0.37	1.82 ± 0.23	1.87 ± 0.37	1.95 ± 0.29
Neutrophil (%)	9.17 ± 1.17	9.50 ± 1.22	10.67 ± 2.42	10.50 ± 1.76	11.00 ± 3.35	13.00 ± 2.28	15.50 ± 2.59 *	15.83 ± 2.71 *
Lymphocyte (%)	87.50 ± 3.67	89.33 ± 1.63	87.17 ± 2.23	84.17 ± 4.45	88.83 ± 4.54	85.00 ± 3.22	83.33 ± 1.86	80.33 ± 8.33 *
Monocyte (%)	0.67 ± 0.52	0.50 ± 0.55	0.50 ± 0.55	0.50 ± 0.55	0.67 ± 0.52	0.67 ± 0.52	0.67 ± 0.82	0.67 ± 0.52
Eosinophil (%)	0.17 ± 0.41	0.17 ± 0.41	0.17 ± 0.41	0.17 ± 0.41	0.50 ± 0.55	0.33 ± 0.52	0.33 ± 0.52	0.33 ± 0.52
Basophil (%)	0.17 ± 0.41	0.00 ± 0.00	0.00 ± 0.00	0.00 ± 0.00	0.00 ± 0.00	0.00 ± 0.00	0.00 ± 0.00	0.00 ± 0.00
Platelet count (×10^3^ cell/µL)	621.33 ± 37.68	614.67 ± 24.86	605.00 ± 37.33	598.67 ± 42.05	645.17 ± 75.28	669.33 ± 89.37	613.33 ± 57.74	593.83 ± 30.50
MCV (fL)	55.50 ± 1.38	55.67 ± 1.86	56.50 ± 0.55	55.83 ± 2.32	57.67 ± 1.21	56.83 ± 0.98	56.83 ± 1.72	56.33 ± 1.37
MCH (pg)	19.00 ± 0.63	18.67 ± 0.52	18.83 ± 0.41	19.00 ± 0.63	20.00 ± 0.63	19.50 ± 0.55	19.50 ± 0.84	19.67 ± 0.52
MCHC (%)	34.00 ± 0.00	33.67 ± 0.52	33.50 ± 0.84	33.83 ± 0.75	34.33 ± 0.52	34.33 ± 0.52	34.50 ± 0.55	34.83 ± 0.75

Values are expressed as mean ± SD. * *p* < 0.05; Six rats per group.

**Table 10:** Blood sugar levels of male and female Wistar rats in the control group were compared with those of the group receiving various concentrations of SL extract for 6 months.

Months	Blood Glucose (mg/dL)							
	Control	Male Wistar Rats			Control	Female Wistar Rats		
		5	25	50		5	25	50
Initial	73.33 ± 3.67	72.50 ± 3.02	72.67 ± 4.89	73.00 ± 3.52	65.33 ± 5.50	65.83 ± 4.58	64.33 ± 4.08	64.17 ± 5.38
1	83.67 ± 3.14	81.50 ± 5.65	75.50 ± 2.88 *	71.67 ± 6.28 *	66.83 ± 1.60	68.00 ± 5.14	65.33 ± 7.84	62.17 ± 5.12
2	68.17 ± 3.71	71.00 ± 3.95	67.67 ± 6.86	66.83 ± 7.36	67.83 ± 7.05	61.17 ± 6.46	65.17 ± 5.91	64.83 ± 7.99
3	73.33 ± 3.88	71.33 ± 5.72	68.33 ± 2.66	69.17 ± 4.88	62.67 ± 3.83	60.67 ± 3.39	60.50 ± 5.86	60.67 ± 3.83
4	79.17 ± 6.59	77.83 ± 8.98	68.67 ± 4.13 *	68.17 ± 5.19 *	55.33 ± 6.38	59.00 ± 5.48	58.83 ± 6.55	56.17 ± 2.04
5	70.83 ± 4.92	66.33 ± 7.12	64.17 ± 5.49	63.33 ± 4.23 *	61.17 ± 8.68	60.50 ± 5.39	60.00 ± 10.08	63.67 ± 6.47
6	72.50 ± 3.62	67.83 ± 4.26	70.67 ± 5.72	63.67 ± 4.76 *	63.67 ± 4.32	62.17 ± 5.08	60.67 ± 5.65	64.33 ± 6.12

Values are expressed as mean ± SD. * *p* < 0.05; Six rats per group.

## Data Availability

The original contributions presented in this study are included in the article. Further inquiries can be directed to the corresponding author.

## Supplementary Materials

The following supporting information can be downloaded at: https://www.mdpi.com/article/10.3390/ijms27020577/s1 [90,91,92,93,94,95].

## References

[1] Wong W.L., Su X., Li X., Cheung C.M., Klein R., Cheng C.Y., Wong T.Y. (2014). Global prevalence of age-related macular degeneration and disease burden projection for 2020 and 2040: A systematic review and meta-analysis. Lancet Glob. Health.

[2] Ranard K.M., Jeon S., Mohn E.S., Griffiths J.C., Johnson E.J., Erdman J.W. (2017). Dietary guidance for lutein: Consideration for intake recommendations is scientifically supported. Eur. J. Nutr..

[3] Beatty S., Boulton M., Henson D., Koh H.H., Murray I.J. (1999). Macular pigment and age related macular degeneration. Br. J. Ophthalmol..

[4] Junghans A., Sies H., Stahl W. (2001). Macular pigments lutein and zeaxanthin as blue light filters studied in liposomes. Arch. Biochem. Biophys..

[5] Age-Related Eye Disease Study 2 (2013). Lutein + zeaxanthin and omega-3 fatty acids for age-related macular degeneration: The Age-Related Eye Disease Study 2 (AREDS2) randomized clinical trial. Jama.

[6] ŠIvel M., Kracmar S., Fišera M., Klejdus B., Kubáň V. (2014). Lutein Content in Marigold Flower (*Tagetes erecta* L.) Concentrates used for Production of Food Supplements. Czech J. Food Sci..

[7] Boon C.S., McClements D.J., Weiss J., Decker E.A. (2010). Factors influencing the chemical stability of carotenoids in foods. Crit. Rev. Food Sci. Nutr..

[8] Yao Y., Lin J.J., Chee X.Y.J., Liu M.H., Khan S.A., Kim J.E. (2021). Encapsulation of Lutein via Microfluidic Technology: Evaluation of Stability and In Vitro Bioaccessibility. Foods.

[9] Liu Y., Ma L., Zhang Q., Liu Y., Li D. (2024). Construction of fatty acid-ovalbumin binary complexes to improve the water dispersibility, thermal/digestive stability and bioaccessibility of lutein: A comparative study of different fatty acids. Int. J. Biol. Macromol..

[10] Dursun Capar T., Yalcin H. (2024). Conjugation prepared by wet-Maillard reactions improves the stability and properties of lutein and lycopene loaded nanoparticles. J. Food Sci. Technol..

[11] Jouni Z.E., Wells M.A. (1996). Purification and Partial Characterization of a Lutein-binding Protein from the Midgut of the Silkworm *Bombyx mori**. J. Biol. Chem..

[12] Liu S., Zhang Q., Zhou H., Zhang B., Yu M., Wang Y., Liu Y., Chai C. (2024). The Potential of Natural Carotenoids-Containing Sericin of the Domestic Silkworm *Bombyx mori*. Int. J. Mol. Sci..

[13] Li B., Vachali P., Frederick J.M., Bernstein P.S. (2011). Identification of StARD3 as a lutein-binding protein in the macula of the primate retina. Biochemistry.

[14] Bernstein P.S., Li B., Vachali P.P., Gorusupudi A., Shyam R., Henriksen B.S., Nolan J.M. (2016). Lutein, zeaxanthin, and meso-zeaxanthin: The basic and clinical science underlying carotenoid-based nutritional interventions against ocular disease. Prog. Retin. Eye Res..

[15] Manupa W., Wongthanyakram J., Jeencham R., Sutheerawattananonda M. (2023). Storage stability and antioxidant activities of lutein extracted from yellow silk cocoons (*Bombyx mori*) in Thailand. Heliyon.

[16] Sung J.H., Jo Y.S., Kim S.J., Ryu J.S., Kim M.C., Ko H.J., Sim S.S. (2013). Effect of Lutein on L-NAME-Induced Hypertensive Rats. Korean J. Physiol. Pharmacol..

[17] Singhrang N., Tocharus C., Thummayot S., Sutheerawattananonda M., Tocharus J. (2018). Protective effects of silk lutein extract from *Bombyx mori* cocoons on β-Amyloid peptide-induced apoptosis in PC12 cells. Biomed. Pharmacother. = Biomed. Pharmacother..

[18] Pongcharoen S., Warnnissorn P., Leŗtkajornsin O., Limpeanchob N., Sutheerawattananonda M. (2013). Protective effect of silk lutein on ultraviolet B-irradiated human keratinocytes. Biol. Res..

[19] Aonsri C., Sutheerawattananonda M., Limpeanchob N. (2015). Protective Effect of Silk Lutein Extract on Hydrogen Peroxide Induced Retinal Pigment Epithelial Cells Damage. Thai J. Pharmacol..

[20] Promphet P., Bunarsa S., Sutheerawattananonda M., Kunthalert D. (2014). Immune enhancement activities of silk lutein extract from *Bombyx mori* cocoons. Biol. Res..

[21] Ministry of Public Health in Thailand (2020). Notification of Ministry of Public Health (No. 414) B.E. 2563 (2020) Re: Standards for Contaminants in Food.

[22] Alves-Rodrigues A., Shao A. (2004). The science behind lutein. Toxicol. Lett..

[23] U.S. Environmental Protection Agency National Primary Drinking Water Regulations. https://www.epa.gov/ground-water-and-drinking-water/national-primary-drinking-water-regulations#Inorganics.

[24] Wong C., Roberts S.M., Saab I.N. (2022). Review of regulatory reference values and background levels for heavy metals in the human diet. Regul. Toxicol. Pharmacol..

[25] Filippini T., Malagoli C., Wise L.A., Malavolti M., Pellacani G., Vinceti M. (2019). Dietary cadmium intake and risk of cutaneous melanoma: An Italian population-based case-control study. J. Trace Elem. Med. Biol..

[26] World Health Organization (2017). Guidelines for Drinking-Water Quality: Fourth Edition Incorporating the First Addendum.

[27] Cabrera Á.J. (2015). Zinc, aging, and immunosenescence: An overview. Pathobiol. Aging Age Relat. Dis..

[28] Manfredi A., Varrà M.O., Zanardi E., Vitellino M., Peloso M., Lorusso P., Ghidini S., Bonerba E., Accurso D. (2025). Dietary exposure assessment to nickel through the consumption of poultry, beef, and pork meat for different age groups in the Italian population. Ital. J. Food Saf..

[29] OECD (2002). Acute Oral toxicity—Acute Toxic Class Method.

[30] Serfilippi L.M., Pallman D.R., Russell B. (2003). Serum clinical chemistry and hematology reference values in outbred stocks of albino mice from three commonly used vendors and two inbred strains of albino mice. Contemp. Top. Lab. Anim. Sci..

[31] Liu Z., Que S., Xu J., Peng T. (2014). Alanine aminotransferase-old biomarker and new concept: A review. Int. J. Med. Sci..

[32] Nguyen M.T., Lian A., Guilford F.T., Venketaraman V. (2025). A Literature Review of Glutathione Therapy in Ameliorating Hepatic Dysfunction in Non-Alcoholic Fatty Liver Disease. Biomedicines.

[33] Munteanu C., Schwartz B. (2023). The Effect of Bioactive Aliment Compounds and Micronutrients on Non-Alcoholic Fatty Liver Disease. Antioxidants.

[34] Siddique A., Kowdley K.V. (2012). Approach to a patient with elevated serum alkaline phosphatase. Clin. Liver Dis..

[35] Thakur S., Kumar V., Das R., Sharma V., Mehta D.K. (2024). Biomarkers of Hepatic Toxicity: An Overview. Curr. Ther. Res..

[36] Sellers R.S., Morton D., Michael B., Roome N., Johnson J.K., Yano B.L., Perry R., Schafer K. (2007). Society of Toxicologic Pathology position paper: Organ weight recommendations for toxicology studies. Toxicol. Pathol..

[37] Kunz R.I., Brancalhão R.M., Ribeiro L.F., Natali M.R. (2016). Silkworm Sericin: Properties and Biomedical Applications. Biomed. Res. Int..

[38] Dzhalilova D.S., Kosyreva A.M., Diatroptov M.E., Zolotova N.A., Tsvetkov I.S., Mkhitarov V.A., Makarova O.V., Khochanskiy D.N. (2019). Morphological Characteristics of the Thymus and Spleen and the Subpopulation Composition of Lymphocytes in Peripheral Blood during Systemic Inflammatory Response in Male Rats with Different Resistance to Hypoxia. Int. J. Inflam..

[39] Nidhi B., Baskaran V. (2013). Acute and subacute toxicity assessment of lutein in lutein-deficient mice. J. Food Sci..

[40] Harikumar K.B., Nimita C.V., Preethi K.C., Kuttan R., Shankaranarayana M.L., Deshpande J. (2008). Toxicity profile of lutein and lutein ester isolated from marigold flowers (*Tagetes erecta*). Int. J. Toxicol..

[41] Palozza P., Catalano A., Simone R.E., Mele M.C., Cittadini A. (2012). Effect of lycopene and tomato products on cholesterol metabolism. Ann. Nutr. Metab..

[42] Bowen P.E., Herbst-Espinosa S.M., Hussain E.A., Stacewicz-Sapuntzakis M. (2002). Esterification does not impair lutein bioavailability in humans. J. Nutr..

[43] Ghasemi F., Navab F., Rouhani M.H., Amini P., Shokri-Mashhadi N. (2023). The effect of lutein and Zeaxanthine on dyslipidemia: A meta-analysis study. Prostagland. Other Lipid Mediat..

[44] Murillo A.G., Hu S., Fernandez M.L. (2019). Zeaxanthin: Metabolism, Properties, and Antioxidant Protection of Eyes, Heart, Liver, and Skin. Antioxidants.

[45] Wang N., Wang D., Luo G., Zhou J., Tan Z., Du Y., Xie H., Liu L., Yang X., Hao L. (2021). Lutein attenuates excessive lipid accumulation in differentiated 3T3-L1 cells and abdominal adipose tissue of rats by the SIRT1-mediated pathway. Int. J. Biochem. Cell Biol..

[46] Cheng J., Liu D., Zhao J., Li X., Yan Y., Wu Z., Wang H., Wang C. (2019). Lutein attenuates oxidative stress and inhibits lipid accumulation in free fatty acids-induced HepG2 cells by activating the AMPK pathway. J. Funct. Foods.

[47] Alvi S.S., Iqbal D., Ahmad S., Khan M.S. (2016). Molecular rationale delineating the role of lycopene as a potent HMG-CoA reductase inhibitor: In vitro and in silico study. Nat. Prod. Res..

[48] Elseweidy M.M., Elawady A.S., Sobh M.S., Elnagar G.M. (2022). Lycopene ameliorates hyperlipidemia via potentiation of AMP-activated protein kinase and inhibition of ATP-citrate lyase in diabetic hyperlipidemic rat model. Life Sci..

[49] Manabe Y., Ueda M., Sugawara T. (2025). β-Carotene Suppresses Lipopolysaccharide-induced Nitric Oxide Production in Microglia via Retinoic Acid Receptor-dependent Mechanisms. J. Oleo Sci..

[50] Lobo G.P., Amengual J., Li H.N., Golczak M., Bonet M.L., Palczewski K., von Lintig J. (2010). Beta,beta-carotene decreases peroxisome proliferator receptor gamma activity and reduces lipid storage capacity of adipocytes in a beta,beta-carotene oxygenase 1-dependent manner. J. Biol. Chem..

[51] Thapa B.R., Walia A. (2007). Liver function tests and their interpretation. Indian. J. Pediatr..

[52] Stocker R., Yamamoto Y., McDonagh A.F., Glazer A.N., Ames B.N. (1987). Bilirubin is an antioxidant of possible physiological importance. Science.

[53] Ramaiah S.K. (2007). A toxicologist guide to the diagnostic interpretation of hepatic biochemical parameters. Food Chem. Toxicol..

[54] Tsao R., Yang R., Young J.C., Zhu H., Manolis T. (2004). Separation of geometric isomers of native lutein diesters in marigold (*Tagetes erecta* L.) by high-performance liquid chromatography–mass spectrometry. J. Chromatogr. A.

[55] Norkus E.P., Norkus K.L., Dharmarajan T.S., Schierle J., Schalch W. (2010). Serum Lutein Response Is Greater from Free Lutein Than from Esterified Lutein during 4 Weeks of Supplementation in Healthy Adults. J. Am. Coll. Nutr..

[56] Gleize B., Tourniaire F., Depezay L., Bott R., Nowicki M., Albino L., Lairon D., Kesse-Guyot E., Galan P., Hercberg S. (2013). Effect of type of TAG fatty acids on lutein and zeaxanthin bioavailability. Br. J. Nutr..

[57] Tocharus C., Prum V., Sutheerawattananonda M. (2024). Oral Toxicity and Hypotensive Influence of Sericin-Derived Oligopeptides (SDOs) from Yellow Silk Cocoons of *Bombyx mori* in Rodent Studies. Foods.

[58] Harris A., Siesky B., Huang A., Do T., Mathew S., Frantz R., Gross J., Januleviciene I., Verticchio Vercellin A.C. (2019). Lutein Complex Supplementation Increases Ocular Blood Flow Biomarkers in Healthy Subjects. Int. J. Vitam. Nutr. Res..

[59] Tukhovskaya E.A., Ismailova A.M., Perepechenova N.A., Slashcheva G.A., Palikov V.A., Palikova Y.A., Rzhevsky D.I., Rykov V.A., Novikova N.I., Dyachenko I.A. (2024). Development and Worsening of Hypertension with Age in Male Wistar Rats as a Physiological Model of Age-Related Hypertension: Correction of Hypertension with Taxifolin. Int. J. Mol. Sci..

[60] Wadley A.J., Veldhuijzen van Zanten J.J., Aldred S. (2013). The interactions of oxidative stress and inflammation with vascular dysfunction in ageing: The vascular health triad. Age.

[61] Hajizadeh-Sharafabad F., Ghoreishi Z., Maleki V., Tarighat-Esfanjani A. (2019). Mechanistic insights into the effect of lutein on atherosclerosis, vascular dysfunction, and related risk factors: A systematic review of in vivo, ex vivo and in vitro studies. Pharmacol. Res..

[62] Wang S., Wang M., Zhang S., Zhao L. (2014). Oxidative stress in rats with hyperhomo-cysteinemia and intervention effect of lutein. Eur. Rev. Med. Pharmacol. Sci..

[63] Han H., Cui W., Wang L., Xiong Y., Liu L., Sun X., Hao L. (2015). Lutein prevents high fat diet-induced atherosclerosis in ApoE-deficient mice by inhibiting NADPH oxidase and increasing PPAR expression. Lipids.

[64] Varlamov O., Bethea C.L., Roberts C.T. (2014). Sex-specific differences in lipid and glucose metabolism. Front. Endocrinol..

[65] Wang Z., Yuexin Y., Xiang X., Zhu Y., Men J., He M. (2010). Estimation of the normal range of blood glucose in rats. Wei Sheng Yan Jiu = J. Hyg. Res..

[66] Gupte A.A., Pownall H.J., Hamilton D.J. (2015). Estrogen: An emerging regulator of insulin action and mitochondrial function. J. Diabetes Res..

[67] Le May C., Chu K., Hu M., Ortega C.S., Simpson E.R., Korach K.S., Tsai M.J., Mauvais-Jarvis F. (2006). Estrogens protect pancreatic beta-cells from apoptosis and prevent insulin-deficient diabetes mellitus in mice. Proc. Natl. Acad. Sci. USA.

[68] Franconi F., Seghieri G., Canu S., Straface E., Campesi I., Malorni W. (2008). Are the available experimental models of type 2 diabetes appropriate for a gender perspective?. Pharmacol. Res..

[69] Sharavana G., Joseph G.S., Baskaran V. (2017). Lutein attenuates oxidative stress markers and ameliorates glucose homeostasis through polyol pathway in heart and kidney of STZ-induced hyperglycemic rat model. Eur. J. Nutr..

[70] Kim D.-S., Kang S., Moon N.-R., Shin B.-K., Park S. (2024). Zeaxanthin and Lutein Ameliorate Alzheimer’s Disease-like Pathology: Modulation of Insulin Resistance, Neuroinflammation, and Acetylcholinesterase Activity in an Amyloid-β Rat Model. Int. J. Mol. Sci..

[71] Reagan-Shaw S., Nihal M., Ahmad N. (2008). Dose translation from animal to human studies revisited. Faseb J..

[72] Chew E.Y., Clemons T., SanGiovanni J.P., Danis R., Domalpally A., McBee W., Sperduto R., Ferris F.L. (2012). The Age-Related Eye Disease Study 2 (AREDS2): Study design and baseline characteristics (AREDS2 report number 1). Ophthalmology.

[73] Sutheerawattananonda M., Kaewkumsan P., Limpeanchob N., Kanthalert D. (2015). Method for Extracting Silk Extract Containing Lutein. U.S. Patent.

[74] International Organization for Standardization, International Electrotechnical Commission (2005). General Requirements for the Competence of Testing and Calibration Laboratories (ISO/IEC Standard No. 17025:2005). https://www.iso.org/obp/ui/#iso:std:iso-iec:17025:ed-2:v1:en.

[75] U.S. Environmental Protection Agency (1996). Method 3052: Microwave Assisted Acid Digestion of Siliceous and Organically Based Matrices.

[76] Food and Drug Administration (2017). Bacteriological Analytical Manual, Chapter 4, Enumeration of Escherichia coli and the Coliform Bacteria.

[77] (2017). Microbiology of the Food Chain—Horizontal Method for the Detection and Enumeration of Listeria Monocytogenes and of *Listeria* spp.—Part 1: Detection Method.

[78] (2017). Microbiology of the Food Chain—Horizontal Method for the Detection, Enumeration and Serotyping of Salmonella—Part 1: Detection of *Salmonella* spp..

[79] Food and Drug Administration (2016). Bacteriological Analytical Manual, Chapter 12, Staphylococcus aureus.

[80] Food and Drug Administration (2001). Bacteriological Analytical Manual, Chapter 3, Aerobic Plate Count.

[81] (2018). Foods of Plant Origin—Multimethod for the Determination of Pesticide Residues Using GC- and LC-Based Analysis Following Acetonitrile Extraction/Partitioning and Clean-Up by Dispersive SPE—Modular QuEChERS-Method.

[82] AOAC (1996). Moisture and volatile matter in oils and fats (926.12-1926). Official Methods of Analysis.

[83] Li C., Wang Z., Li G., Wang Z., Yang J., Li Y., Wang H., Jin H., Qiao J., Wang H. (2020). Acute and repeated dose 26-week oral toxicity study of 20(S)-ginsenoside Rg3 in Kunming mice and Sprague-Dawley rats. J. Ginseng Res..

[84] Wang D., Zhang W., Ju J.-X., Wang L.-J., Huang R.-Y., Xu Y.-F., Zhang H.-L., Qi J.-L. (2021). Gender differences in acute toxicity, toxicokinetic and tissue distribution of amphotericin B liposomes in rats. Toxicol. Lett..

[85] Hubrecht R.C., Carter E. (2019). The 3Rs and Humane Experimental Technique: Implementing Change. Animals.

[86] Tocharus C., Saelim J., Sutheerawattananonda M. (2025). Consumption of Sericin Enhances the Bioavailability and Metabolic Efficacy of Chromium Picolinate in Rats. Int. J. Mol. Sci..

[87] Musa A.H., Gebru G., Debella A., Makonnen E., Asefa M., Woldekidan S., Lengiso B., Bashea C. (2022). Chronic (52-week) oral toxicity study of herbal tea of Moringa stenopetala and Mentha spicata leaves formulation in Wistar albino rats. Int. J. Pharm. Sci. Dev. Res..

[88] Chavalittumrong P., Chivapat S., Attawish A., Bansiddhi J., Phadungpat S., Chaorai B., Butraporn R. (2004). Chronic toxicity study of Portulaca grandiflora Hook. J. Ethnopharmacol..

[89] Crissman J.W., Goodman D.G., Hildebrandt P.K., Maronpot R.R., Prater D.A., Riley J.H., Seaman W.J., Thake D.C. (2004). Best practices guideline: Toxicologic histopathology. Toxicol. Pathol..

[90] Hair H., Macleod M.R., Sena E.S. (2019). A randomised controlled trial of an Intervention to Improve Compliance with the ARRIVE guidelines (IICARus). Res. Integr. Peer Rev..

[91] Tihanyi D.K., Szijarto A., Fülöp A., Denecke B., Lurje G., Neumann U.P., Czigany Z., Tolba R. (2019). Systematic Review on Characteristics and Reporting Quality of Animal Studies in Liver Regeneration Triggered by Portal Vein Occlusion and Associating Liver Partition and Portal Vein Ligation for Staged Hepatectomy: Adherence to the ARRIVE Guidelines. J. Surg. Res..

[92] Zhao B., Jiang Y., Zhang T., Shang X., Zhang W., Hu K., Chen F., Mei F., Gao Q., Zhao L. (2020). Quality of interventional animal experiments in Chinese journals: Compliance with ARRIVE guidelines. BMC Vet. Res..

[93] Han S., Olonisakin T.F., Pribis J.P., Zupetic J., Yoon J.H., Holleran K.M., Jeong K., Shaikh N., Rubio D.M., Lee J.S. (2017). A checklist is associated with increased quality of reporting preclinical biomedical research: A systematic review. PLoS ONE.

[94] Chatzimanouil M.K.T., Wilkens L., Anders H.-J. (2019). Quantity and Reporting Quality of Kidney Research. J. Am. Soc. Nephrol..

[95] Leung V., Rousseau-Blass F., Beauchamp G., Pang D.S.J. (2018). ARRIVE has not ARRIVEd: Support for the ARRIVE (Animal Research: Reporting of in vivo Experiments) guidelines does not improve the reporting quality of papers in animal welfare, analgesia or anesthesia. PLoS ONE.

